# Enhancing medication management in hemodialysis patients: Exploring the impact of patient-centered pharmacist care and motivational interviewing

**DOI:** 10.1371/journal.pone.0300499

**Published:** 2024-05-21

**Authors:** Ganesh Sritheran Paneerselvam, Lee Kwing Chin Kenneth, Raja Ahsan Aftab, Roland Gamini Sirisinghe, Pauline Siew Mei Lai, Soo Kun Lim

**Affiliations:** 1 School of Pharmacy, Faculty of Health and Medical Science, Taylor’s University, Selangor, Malaysia; 2 Department of Clinical Pharmacy and Pharmacy Practice, Faculty of Pharmacy, University of Malaya, Kuala Lumpur, Malaysia; 3 Department of Primary Care Medicine, Faculty of Medicine, University of Malaya, Kuala Lumpur, Malaysia; 4 Department of Medicine, Faculty of Medicine, University of Malaya, Kuala Lumpur, Malaysia; AIIMS Jodhpur: All India Institute of Medical Sciences - Jodhpur, INDIA

## Abstract

**Background:**

Patients on hemodialysis (HD) often uses several medications, making them highly susceptible to medication-related problems (MRP) thereby leading to medication nonadherence. Therefore, an innovative pharmaceutical care strategy incorporating drug therapy optimization (DTO) and motivational interviewing (MI) can mitigate medication-related problems and optimize patient care.

**Aims and objective:**

The objective of this study is to assess the efficacy of pharmacist led interventions in utilizing DTO and MI techniques in managing medication related problems among patients undergoing hemodialysis.

**Method and design:**

A12-months, cross sectional prospective study was conducted among 63 End Stage Renal Disease (ESRD) patients on HD. DTO was conducted by the pharmacist to identify the MRP by reviewing complete medication list gathered from patient interview and medical records. All MRPs was classified using the PCNE classification version 9.00 and medication issues, that require patient involvement were categorized as patient-related, while those that necessitate physician intervention were classified as physician-related. The DTO was performed at the baseline, 6-month and at the final month of the study. Identified medication issues were communicated to the site nephrologist and was tracked during next follow up. Whereas MI was conducted physically at Month-3 and via telephone on month-6 and month-9 to address patient related medication issues.

**Results:**

Mean age of the study population was 48.5±14 years. While the mean number of prescribed medications was 8.1±2 with 57% of the patients taking more than 5 types of medication. After 12 months of pharmacist intervention using DTO and MI, a mean reduction in MRP was observed for both patient-related and physician-related MRPs across three time series. However, further analysis using repeated measure ANOVA revealed that the reduction in patient-related MRPs was statistically significant [F(1.491, 92.412) = 60.921, p < 0.05], while no statistically significant difference was detected in physician-related MRPs [F(2, 124) = 2.216, P = 0.113].

**Conclusion:**

Pharmaceutical care service through DTO and MI can effectively reduce and prevent drug-related issues to optimize medication therapy among HD patients.

## Introduction

Hemodialysis (HD) is the primary form of renal replacement therapy for patients with end-stage renal disease (ESRD). According to the Malaysian Dialysis and Transplant Registry, the number of new dialysis patients has been increasing yearly, with 8,431 new cases reported in 2018, a 5% increase from the previous year [1]. While advances in dialysis treatment have improved the survival rates of ESRD patients, mortality rates among those on chronic dialysis remain high due to the presence of inflammation, undernutrition, and heart-related problems [2, 3].

Patients undergoing HD are at high risk of polypharmacy due to their need for multiple medications to manage chronic comorbidities. This prevalence of polypharmacy among HD patients leads to medication-related problems (MRP), with an estimated incidence of one MRP identified for every 15.2 drug exposures [4]. MRP in ESRD patients on dialysis can result in morbidity, nonadherence to prescribed oral medications, and poor compliance, which is a social issue influenced by patients’ beliefs and myths about medication. Complex interventions that require lifestyle modifications, which are worthwhile to address patients’ beliefs and intentions to perform actions, are needed. Motivational interviewing is a patient centered counseling technique that focuses on enhancing patient motivation and confidence to make positive changes in their health behavior [5]. It is used to promote autonomy for self-direction based on patient goals and values, and it is widely used in medical settings for counseling and psychotherapy [6]. Similarly, Drug therapy optimization involves reviewing patient’s medications to identify potential drug related problems and optimize their pharmacotherapy. This allows adjusting patient medication dosage, changing medication or even discontinuing unnecessary medication.

A combination of drug therapy optimization and motivational interviewing aids in developing a comprehensive patient care approach to address both medication related problems and patient behavior thereby leading to improve medication adherence and optimized patient outcomes among hemodialysis patients. This forms the basis of our study hence the current study was designed to evaluate the effectiveness of DTO and MI interventions in resolving medication issues and optimizing patient care among HD patients.

## Materials and methods

### Study setting and population

The study was conducted at the Hemodialysis unit, Hospital Kuala Lumpur (HKL) and the Hemodialysis Affiliated Centers of the University Malaya Medical Centre (UMMC). These hospitals offer dialysis treatment with a modern and fully equipped center with specialized nephrologist and well-trained staffs. The study was conducted for a period of 12 months among HD patients at these units. Patients were eligible for inclusion if they were aged 18 years and above, undergoing HD treatment (thrice a week) for at least 3 months, able to communicate in English or Malay and willing to give their informed consent. Patients with major surgical interventions in the previous three months, or have malignancies, cognitive impairment, dementia, active psychosis, or major hearing impairment, or were pregnant or breastfeeding, were excluded.

### Study design

This hospital-based prospective observational study was used to measure the impact of pharmacist led intervention by DTO and MI among recruited HD patients. The sample size calculation was performed using Power and Sample Size Calculation Version 3.0 software. The software calculation utilized key parameters, including the confidence level (95%), which corresponds to a Z-statistic of 1.96. The significance level (α) was set at 0.05, and the type II error (β) was considered. The effect size (ES), calculated as the difference in population means (δ) divided by the standard deviation of the difference in response of matched pairs (σ), was crucial for determining the sample size.

Using δ = 0.9 and σ = 1.84, obtained from a similar previous study [7], the resulting sample size was 56 subjects needed to confidently reject the null hypothesis. To account for potential dropout or incomplete data, a conservative estimate was made by adding 25% to the calculated sample size. This compensated for potential attrition, yielding an approximate sample size of 70 respondents. Additionally, considering a dropout rate of 25%, 14 patients were inclusively added, bringing the total to 70 patients overall. This comprehensive approach ensures that the study maintains robust statistical power and accommodates any unforeseen variations in participant retention. The study employed an internal comparison design where all the recruited patients based on the predefined inclusion criteria were observed within the cohort itself over time (baseline, 6^th^ month, final follow up).

**Patient selection.** List of all HD patients was obtained from the respective HD units. A simple random sampling technique using research randomizer, was used to recruit potential patients [8]. A written, informed consent was taken from the selected patients prior to participation. All demographic data of the selected patients such as age, gender, past medical and medication history, dialysis history, medications prescribed [name, dose, frequency, route, duration of the drug] were obtained from electronic health records.

### Instruments used

All medication related problems will be classified using the PCNE classification version 9.00 [9]. According to PCNE, medication related problems will be categorised into nine primary domains for DRP causes. The identified MRPs in our study were clearly separated into physician- and patient-related categories. Problems with medications that necessitate the involvement of the patient were categorized as being patient-related, whereas problems that call for the intervention of the physician were categorized as being physician-related.

### Interventions

Two interventions were provided in this current study. They are drug therapy optimization and motivational interviewing.

#### a) Drug therapy optimization

DTO is a well-structured method that is utilized to optimize medication use and improve health outcomes among patients. During patient dialysis sessions, the researcher interviewed all the recruited HD patients, verified the actual medication use (including non-prescription medicines) and medication administration pattern. The list was then compared to complete medication history based on the patients’ dispensing data by assessing the medical record available at the dialysis unit. Identified MRPs were classified using the PCNE version 9. Medication issues that require patient involvement were categorized as patient-related, while those that necessitate physician intervention were classified as physician-related. This distinction helps ensure that both patients and physicians were fully engaged in the care process to achieve optimal health outcomes.

#### b) Motivational interviewing

The statement described motivational interviewing as an interactive counseling technique that engaged patients in thinking and talking about their medication-taking behavior. This technique empowered patients with necessary information about their diseases, addressed their beliefs around medication, overcame barriers for nonadherence, and optimized medication use on an individual basis [10]. The approach consisted of four domains used in this study, such as providing information about their diseases, addressing beliefs on the medication, overcoming barriers for non-adherence, and providing specific instructions to optimize the use of each medication. These domains aligned with the MI principles and strategies described by Miller & Rollnick [11]. The MI was performed by the researcher who is a pharmacist. Before the study’s initiation, the researcher received training regarding MI techniques from a professional counselor. The training for MI skills included reading materials, viewing video demonstrations related to MI, and attending discussion with the counselor. The MI technique in this study was carried out without a structured guideline for the flow of the session, as studies had indicated that the traditional method might produce poorer outcomes [12].

### Data collection procedure

During the initial visit, all the selected patients’ demographic data as well as prescribed medications was retrieved from the respective patient’s medical records. The researcher then performed the DTO at the baseline, 6-month and at the final month of the study. Identified medication issues with complete recommendations were communicated to the site nephrologist via email. The changes done by the site nephrologist was notified to the researcher and was tracked during next follow up.

**Fig 1:**
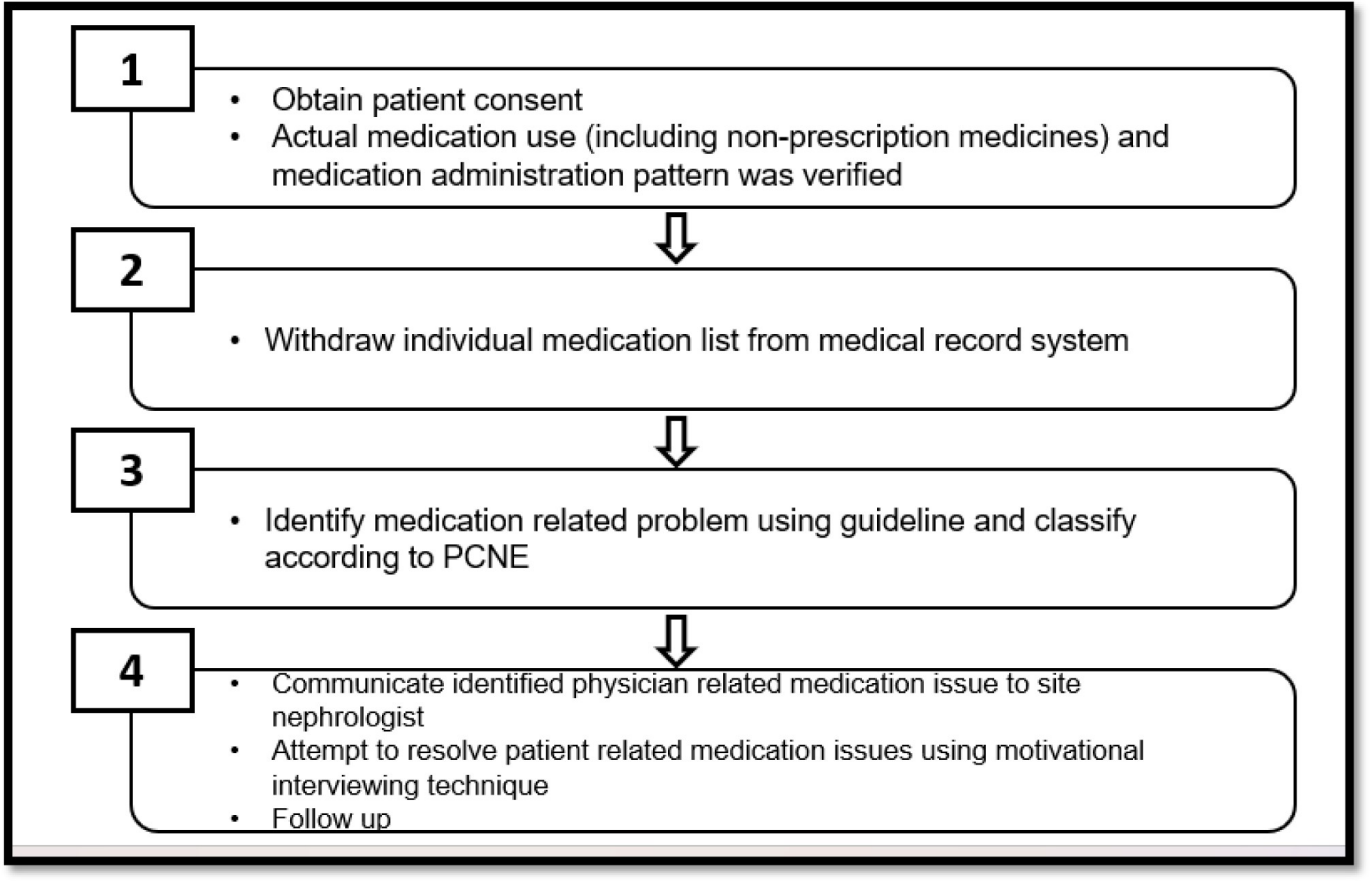
Workflow pharmacist-led pharmaceutical care.

**Fig 2:**
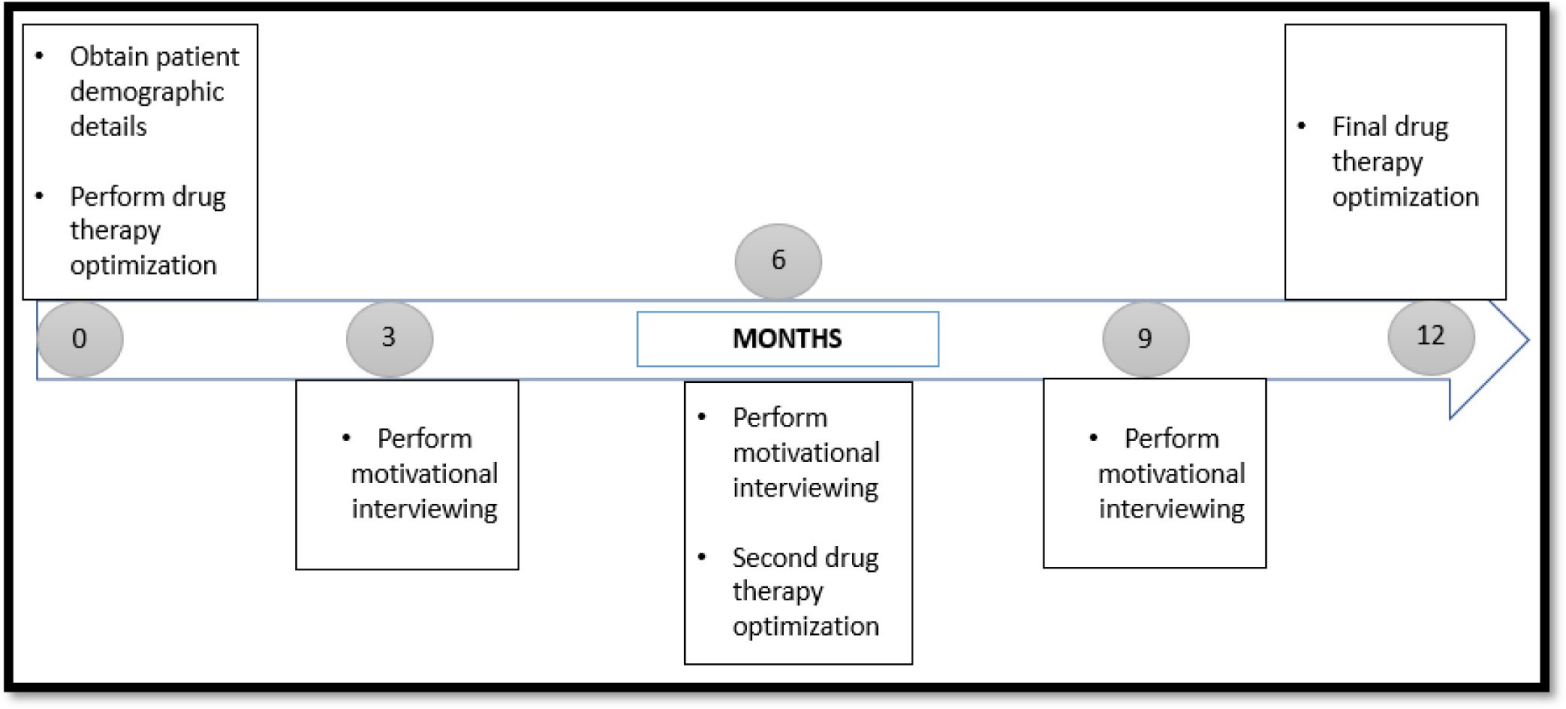
Flow chart summary of study procedure.

While MI was conducted in 3 sessions, started at Month-3 and continued on the month-6 and month-9. Each session was approximately 15 to 20 minutes for each patient. Only the first session will be done face to face (during the patients scheduled dialysis day) to build a good relationship with the patient. While the second and third session was conducted via telephone call. This method has proven its efficacy from previous research [13, 14] and ensures safety for both the patients as well as the researcher. While telephone counselling has its advantages, it also introduces hurdles and makes care less accessible for some people. Previously reported barriers included a lack of access to devices, equipment, slow internet connection and noisy environment as well as privacy and confidentiality concerns [15, 16]. While no major issues were encountered during the telephonic counselling in our study. Occasionally, patients requested a call back at their preferred time, which we accommodated to ensure their comfort and participation. Three sessions were chosen to motivate and improve their medication taking behavior. A comprehensive depiction of the workflow that characterizes the pharmacist-led pharmaceutical care process in this study is presented in Fig 1. While a visual representation in the form of a flow chart, encapsulating the activities carried out by the researcher by month is shown in Fig 2. This concise yet informative diagram offers a clear and accessible overview of the procedural journey undertaken during the study.

### Statistical analysis

Recruited HD patients’ demographic data such as age, gender, marital status, employment, duration of dialysis, number of medications, type of comorbidity and class of medications, were expressed in frequency and percentage, to gain a comprehensive understanding of participant characteristics. While the number of patient and physician related MRPs were shown in frequency and percentage for the three timelines, baseline, month-6 and final respectively. To investigate the impact of pharmacist intervention on the changes in the number of MRP, a repeated-measures ANOVA was employed. This method enabled a meticulous examination of variations over multiple time points, aligning with the longitudinal nature of our study. The utilization of this statistical test facilitated a comprehensive understanding of the intervention’s effects on the observed changes in MRP, contributing to the overall robustness of our research findings. In repeated-measures ANOVA test, sphericity is an important assumption where the variances of the differences between all possible pairs of within-subject conditions should be equal. Mauchly’s test of sphericity was used to evaluate whether the sphericity assumption has been violated. If sphericity is violated, then the variance calculations changes and increases in the type 1 error rate. Therefore, corrections need to be done to modify the degree of freedom so that the F-ratio can be obtained. The correction can be done by using Greenhouse-Geisser correction. All statistical analyses were performed by SPSS Version 27 software [17]. The results had a confidence interval of 95%, p < 0.05 was set as significant level.

## Results

Mean age of the study population was 48.5±14 years, 57.1% of them were female. About 81% of the patients were married and almost three quarter of them were jobless. While the mean number of medications was 8.1±2 with 57% of the patients taking more than 5 types of medication. The mean dialysis duration was 7.7±6 years. In our study, the most reported comorbidity was hypertension, 39(33%) followed by diabetes mellitus 20(16.4%) and hyperlipidemia 12(9.8%). In total, there were around 539 medications taken by the HD patients. The “class of medication” (Table 1), refers to categorization of the medications based on their therapeutic classes. Most patients receive medication for anemia (32.5%), followed by 16.7% antihypertensive medication, 13.6% phosphate binder and others as listed in Table 1.

**Table 1:** Socio and clinical demographic characteristics of patients enrolled in the study (n = 63).

Characteristic	n (%)	Mean(±SD)
**Age**		48.5±14
< 65	54(85.7)	
≥ 65	9(14.3)	
**Gender**		
Male	27(42.9)	
Female	36(57.1)	
**Marital status**		
Single	11(17.5)	
Married	51(81)	
Separated	1(1.6)	
**Employment**		
Yes	16(25.4)	
No	47(74.6)	
	
**No of medications**		8.1±2
< 5	6(9.5)	
≥5	57(90.5)	
**Duration of dialysis**		7.7±6
<5	32(50.8)	
≥5	31(49.2)	
**Types of comorbidly**		
Hypertension	39(33)	
Diabetes mellitus	20(16.4)	
Hyperlipidemia	12(9.8)	
Others • thyroid problem • ischemic heart disease • systemic lupus erythematosus • stroke • asthma • hepatitis B • hepatitis C remainder	5(4.1) 12(9.8) 4(3.3) 2(1.6) 4(3.3) 5(4.1) 4(3.3) 15(12.3%)	
**Class of medication**		
Antihypertensive	90(16.7)	
Antidiabetic	15(2.7)	
Antihyperlipidemic	35(6.4)	
Iron	29(5.5)	
Anemia	175(32.5)	
Proton pump inhibitor	15(2.7)	
Phosphate binder	72(13.6)	
Vitamin D	62(11.5)	
Antiplatelet	21(3.8)	
Asthmatic	12(2.2)	
Others	13(2.4)	

### Impact of pharmacist led intervention via MI and DTO on patient related MRP

The type and frequency of different types of MRPs identified among the recruited HD patients at different time points: baseline, 6-month follow-up, and final assessment was summarized in table 2. At baseline, 123 (number) patient related MRPs were identified, with the most common being “patients taking less medication than prescribed or not taking it at all”. At the 6-month follow-up, there was a reduction of 69% in patient-related MRPs, with a total of 39 MRPs identified. The most common patient-related problem was still “patients taking less medication than prescribed”, but there was a reduction of 52 cases compared to baseline. At the final follow-up, a total of 21 patient related MRPs were identified. There was a significant drop of 80.8% from the baseline level and 42.3% from the 6-month follow-up in the number of identified MRPs. Medication class with the highest frequency of MRPs at baseline were phosphate binder (n = 41), which reflects to “patients consumed less than prescribed dose” DRP category. This was similar in 6-months follow up but shown a reduction of 67%.

Overall, Table 2 shows a gradual decrease in number of patient related MRP after the pharmacist involvement. The mean values in Table 3 shows that patient associated MRPs detected over time. Further analysis using repeated measure ANOVA was done to determine the impact of pharmacist intervention on patient associated MRPs showed there was a statistically significant difference in the mean MRP for patients between time points, baseline, 6- month, and final follow-up (F (1.491,92.412) = 60.921, p<0.05).

**Table 2:** Types and frequency of patients related MRPs identified among recruited HD patients.

Frequency of follow up	Baseline n(%)	6-month n(%)	Final n(%)
**Domains**			
**Patient takes less drug than prescribed/ does not take drug at all**	78 (63.4)	26(66.7)	15(71.4)
**Patient takes more drug than prescribed**	8(6.5)	2(5.1)	1(4.8)
**Patient decides to use unnecessary drug**	10(8.1)	1(2.6)	0(0)
**Patient takes food that interacts**	0(0)	2(5.1)	0(0)
**Inappropriate timing or dosing intervals**	25(20.4)	6(15.4)	2(9.5)
**Patient physically unable to use drug/form as directed**	0(0)	0(0)	1(4.8)
**Patient unable to understand instructions properly**	2(1.6)	2(5.1)	2(9.5)
**TOTAL**	123	39	21

**Table 3:** A repeated-measures ANOVA for overall patient associated medication related problems from baseline till the final follow up.

Outcome	Baseline (n = 63)	6-month (n = 63)	Final (n = 63)	Greenhouse Geiser and P value
**Patient associated MRP**	1.93±1.34	0.65±0.6	0.33±0.6	F(1.491,92.412) = 60.921, P<0.05, η^2^ = 0.49

In summary, a significant reduction in patient associated MRPs can be seen in Table 3.

### Impact of pharmacist led intervention via MI and DTO on physician related MRP

As shown in Table 4, the study identified a total of 92 errors in drug therapy at the baseline, with prescribing drugs at a low dose and not providing complete drug treatment for an existing indication being the most common types of medication-related problems (MRPs) in the dose and drug selection domains, respectively. During the 6-month follow-up, 84 physicians-related MRPs occurred, with underdosing and overprescribing being the most common issues in the dose selection domain, and “lack of indication” and “incomplete treatment” in the drug selection domain. At the end of the follow-up, 71 physicians related MRPs were found, with “incomplete pharmacological treatment” being the largest category of MRP. For physician related, inadequacy targeting the anemia correction with folic acid and EPO was the highest recorded class of medication.

**Table 4:** Types and frequency of physicians related MRPs identified among recruited HD patients.

Frequency of follow up	Baseline	6-month	FINAL
**Domains**			
**Dose selection**			
Drug dose too low	**14(15.2)**	**19(22.6)**	**13(18.3**)
Drug dose of a single active ingredient too high	7(7.6)	13(15.5)	7(9.9)
Dosage regimen too frequent	11(12.0)	12(14.3)	11(15.5)
Dose timing instructions wrong, unclear or missing	2(2.2)	1(1.2)	1(1.4)
**Drug selection**			
No indication for drug	12(13.0)	14(16.7)	6(8.5)
Inappropriate combination of drugs, or drugs and herbal medications, or drugs and dietary supplements	1(1.1)	1(1.2)	1(1.4)
Too many different drugs/active ingredients prescribed for indication	0	1(1.2)	1(1.4)
No or incomplete drug treatment in spite of existing indication	24(26.1)	18(21.4)	27(38.0)
**Treatment duration**			
Duration of treatment too long	0(0)	1(1.2)	0(0)
**Other**			
No or inappropriate outcome monitoring	21(22.8)	4(4.7)	4(5.6)
**TOTAL**	92	84	71

Table 4 indicates inconsistencies in the number of physician-related MRPs. The outcome of pharmacist-led intervention on physician related MRPs were displayed in Table 5. The mean number of physician-associated MRPs decreased from 1.4±1.1 at baseline to 1.3±0.9 at 6-month and further to 1.1±1.0 at the final stage, indicating a reduction in MRPs over time. However, statistical analysis using a repeated-measures ANOVA revealed a non-significant effect of time on physician-associated MRPs, F (2,124) = 2.216, P = 0.113, η^2^ = 0.035), suggesting that the reduction in MRPs over time was not statistically significant.

**Table 5:** A repeated-measures ANOVA for overall physician associated medication related problems from baseline till the final follow up.

Outcome	Baseline (n = 63)	6-month (n = 63)	Final (n = 63)	Sphericity Assumed and P value
**Physician associated MRP**	1.4±1.1	1.3±0.9	1.1±1.0	F(2,124) = 2.216, P = 0.113, η^2^ = 0.035

In summary, an insignificant reduction in physician related MRPs can be seen in Table 5.

Table 6 shows a progressive reduction in the number of physician and patient related MRPs over time. The percentage reductions from baseline at 6 months and the final time point indicate the extent of improvement in managing MRPs identified among recruited HD patients. The intervention appears to have a positive impact on reducing MRPs, with a more substantial reduction observed at the final assessment compared to the 6-month follow-up.

**Table 6:** Total patient and physician related MRPs.

Timeline	No of physician and patient related MRP	Percentage reduction from baseline
Baseline	215	-
6-month	123	42.8
Final	92	57.2

Table 7 presents the mean MRPs detected, encompassing both patient and physician-related factors across the three timelines. Following the pharmacist intervention, a notable reduction in the mean number of identified MRPs was evident from baseline to the final follow-up. These findings offer compelling evidence of statistically significant changes in the parameter, observed not only from baseline to both the 6-month and final month but also between the 6-month and final month. The consistent decrease in means over time serves as a strong indicator of improvement in the studied variable.

**Table 7:** Total patient and physician related MRPs.

Timeline	Baseline (n = 63) Mean (SD)	6-month (n = 63) Mean (SD)	Final (n = 63) Mean (SD)	P- value
Baseline vs 6-month	3.4(1.96)	1.9(1.1)		0.012
Baseline vs final month	3.4(1.96)		1.4(1.2)	0.001
6-month vs final month		1.9(1.1)	1.4(1.2)	0.028

## Discussion

In this study, reduction in patient related MRPs from baseline till the end of the study was statistically significant which proves that pharmacy-based MI and DTO have an important impact on the care of HD by reducing the number of MRPs. A systematic review study reported that four of eight studies using MI, showed a statistically significant effect on primary outcomes like medication adherence, hospital readmissions, and emergency department visits while other studies showed improvement in mean scores [18]. Most of the studies report the outcomes as improvement in adherence after the MI, but in this study overall patient related MRPs which includes inappropriate medication taking, improper dosing or administration and others, shows a decrease after the pharmacist MI. This technique not only satisfies the pharmacist as the provider but also the patient because they feel more understood and supported by having a dynamic relationship with the provider [19].

The most common medication that patient forget taking as prescribed was phosphate binder. Similar to previous study, almost 74% of ESRD patients thought to be noncompliance to the phosphate binder medication [20]. Lack of belief, forgetfulness, social embarrassment caused by taking tablets with others, lack of knowledge, inability to eat together with food, and frequent dosing intervals were the patient related factors [21–23]. While medication related factors such as side effects, large tablet size, unpalatable taste, were the key contributors to phosphate binder nonadherence [24].

Improper dose selection and no or incomplete medication for existing condition was the most type of MRP recorded in the physician related. The findings were different from another study done by Ismail et al. (2019) among HD patients in Saudi Arabia, that shows drug used without indication was the most type of MRPs [25]. While another study, found that indication without drug was the greatest identified MRP [26]. It can be said the type MRPs identified may differ by country, settings of HD and physician experience. The less acceptance rate might be due to the challenges communicating with other clinical team members owing to inadequate time, lack of clear evidence-based guidelines or studies and conflicts between the role of the pharmacist, physician, and specialists that may also impair deprescribing or medication adjustments [27, 28]. Additionally, physicians were reluctant to accept many of the interventions because they might require referrals to other specialists to reevaluate the individual patient’s requirements. Therefore, a more organized physician-pharmacist cooperation is needed to improve the effectiveness of drug therapy among HD patients.

Folic acid medication was not added to the medication regime for anemia management among recruited HD patients. A study by Garg et al. (2001) conducted among HD patients in Unites States reported that lower level of folic acid has been seen in HD patients due to malnutrition and folate loss through dialysate [29]. Therefore, folic acid intake by HD patients can help to reduce cardiovascular disease by reducing the serum homocysteine levels [30, 31]. Besides that, insufficient anemia management with erythropoietin (EPO) was also observed. Study by Rogg et al (2019) in HD patients at New York, states that managing anaemia with EPO drugs can be difficult because each patient’s reaction to treatment is unique [32]. On the other hand, aggressive dose changes before adequate timing for Hb response to be seen in patient and lack of constant monitoring, will overshoot the Hb target and leads to cardiovascular risk [33]. Since there are benefits and risks in EPO dose adjustments, new methods of precise monitoring and prescribing EPO dose should be done. Overall, findings of current study further strengthen the fact to address medication related problems needs to be addressed at different levels and require multidisciplinary approach in enhancing pharmaceutical care.

### Limitation

The researcher declares some limitations to this study. First, its findings cannot be generalized because they are restricted to a specific sample group. This study needs to be conducted as pre-post due to ethical consideration from study site.

## Conclusions

In conclusion, our study demonstrated a significant reduction in patient-related MRPs from baseline to study completion, highlighting the substantial impact of pharmacy-based MI and DTO interventions on enhancing care for HD patients. These two interventions positively influence medication adherence and fosters patient satisfaction and support. Moreover, through resolving patient related MRPs, it facilitates the modification of self-care and self-management behaviors among HD patients. However, resolving physician related MRPs was still a challenge due to poor coordinated physician-pharmacist collaboration in optimizing drug therapy among HD patients. Nevertheless, its undeniable clinical value as a tool for recognizing and addressing MRPs among these patients remains undeniable.

## Recommendation

To address these challenges, our study emphasizes the necessity of a multidisciplinary approach to tackle medication-related issues comprehensively. Collectively, our findings suggest the pivotal role of integrated interventions and collaborative efforts between pharmacist and physician in achieving comprehensive pharmaceutical care for HD patients, ultimately contributing to enhanced patient outcomes and healthcare quality. Overall, this study gives an idea in redesigning the current method of patient care used in hospital as well increase the confidence of pharmacist role in managing HD patients.

## Supporting information

S1 Checklist STROBE statement—checklist of items that should be included in reports of observational studies. (DOCX)

## Abbreviation

HD: hemodialysis
MRP: medication-related problems
MI: motivational interviewing
DTO: drug therapy optimization
PCNE: Pharmaceutical Care Network Europe
MTAC: Medication Therapy Adherence Clinic
HKL: Hospital Kuala Lumpur
UMMC: University Malaya Medical Centre
ES: Effect Size
ANOVA: Analysis of Variance
EPO: erythropoietin
